# Efficient photocatalysis with graphene oxide/Ag/Ag_2_S–TiO_2_ nanocomposites under visible light irradiation†

**DOI:** 10.1039/c7ra13501g

**Published:** 2018-02-05

**Authors:** Shuang Shuang, Ruitao Lv, Xiaoyang Cui, Zheng Xie, Jian Zheng, Zhengjun Zhang

**Affiliations:** State Key Laboratory of New Ceramics and Fine Processing, School of Materials Science and Engineering, Tsinghua University Beijing 100084 China; Key Laboratory of Advanced Materials (MOE), School of Materials Science and Engineering, Tsinghua University Beijing 100084 China zjzhang@tsinghua.edu.cn; High-Tech Institute of Xi'an Xi'an 710025 China; Department of Chemistry, University of Oslo Sem Sælands Vei 26 0371 Oslo Norway

## Abstract

Lack of visible light response and low quantum yield hinder the practical application of TiO_2_ as a high-performance photocatalyst. Herein, we present a rational design of TiO_2_ nanorod arrays (NRAs) decorated with Ag/Ag_2_S nanoparticles (NPs) synthesized through successive ion layer adsorption and reaction (SILAR) and covered by graphene oxide (GO) at room temperature. Ag/Ag_2_S NPs with uniform sizes are well-dispersed on the TiO_2_ nanorods (NRs) as evidenced by electron microscopic analyses. The photocatalyst GO/Ag/Ag_2_S decorated TiO_2_ NRAs shows much higher visible light absorption response, which leads to remarkably enhanced photocatalytic activities on both dye degradation and photoelectrochemical (PEC) performance. Its photocatalytic reaction efficiency is 600% higher than that of pure TiO_2_ sample under visible light. This remarkable enhancement can be attributed to a synergy of electron-sink function and surface plasmon resonance (SPR) of Ag NPs, band matching of Ag_2_S NPs, and rapid charge carrier transport by GO, which significantly improves charge separation of the photoexcited TiO_2_. The photocurrent density of GO/Ag/Ag_2_S–TiO_2_ NRAs reached to maximum (*i.e.* 6.77 mA cm^−2^*vs.* 0 V). Our study proves that the rational design of composite nanostructures enhances the photocatalytic activity under visible light, and efficiently utilizes the complete solar spectrum for pollutant degradation.

## Abstract 2

The photocatalytic reaction efficiency of GO/Ag/Ag_2_S–TiO_2_ nanorod arrays is 600% higher than that of a pure TiO_2_ sample under visible light.

## Graphical abstract

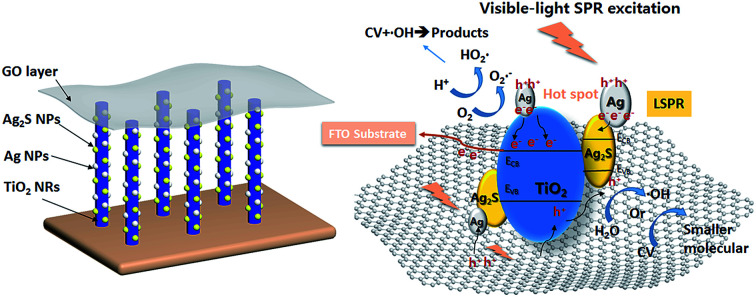

## Introduction

Heterogeneous photocatalysis of pollutants over semiconductor materials has recently emerged as an efficient method for purifying water and air. Among different semiconductors, TiO_2_, usually as n-type semiconductor, is the most widely used photocatalyst due to its excellent photocatalytic activity, non-toxicity and good stability.^1,2^ However, the wide band gap (3.2 eV) of TiO_2_ seriously limits its utilization under visible light which occupies a large portion of the solar spectrum. Till now, different methods such as metal decoration,^3,4^ metallic ion doping,^5^ non-metal doping,^6,7^ and dye sensitization^8,9^ have been proposed to modify and narrow its band gap, and enhance its photocatalytic activity under visible light radiation.^10–12^

Decorating TiO_2_ with metal, as one of the promising methods to develop highly efficient visible light photocatalysts, is becoming attractive. The deposition of metal on TiO_2_, as one of the ways to combine metal with TiO_2_, can greatly improve its photo-efficiency through the Schottky barrier conduction band (CB) electron trapping and consequent longer electron–hole pair lifetime.^3,13^ Hu *et al.*^14^ reported a highly efficient Pt-decorated TiO_2_ which demonstrates enhanced photocatalytic activity for NO_*x*_ oxidation under both UV and visible light irradiation. On the other hand, some noble metals such as Ag and Au, exhibit strong UV-vis absorption due to their plasmon resonance, produced by the collective oscillations of surface electrons.^15^

In other ways, coupling TiO_2_ with different semiconductors is one of common ways to utilize the merit of various semiconductors and enhance the performance of photocatalysts.^16^ Some typical semiconductors like Ag_2_S,^17^ GO,^18^ WO_*x*_,^19^ AgVO_*x*_^20^ and *et al.* Ag_2_S as a typical n-type semiconductor, has low energy bandgap (about 1.0 eV) which enables it to absorb visible and near infrared (NIR) light without the necessity of further doping process. Moreover, quantum dots (QDs) of Ag_2_S has attracted increasing research interest due to their low toxicity, narrow bandgap, and high absorption coefficient.^21^ So, it has been demonstrated that Ag_2_S QDs could perform as a potential excellent visible and NIR photocatalyst.^22^

Graphene oxide (GO) having sufficient reactive oxygen functional groups, is a good candidate for supporting metal or metal oxide particles. The existence of p-conjugation structure and oxygen groups increases the photosensitivity of GO under visible light irradiation, and make it hydrophilic with good electronic performance. The electron energy gap of GO can be adjusted from 3.5 eV to 2.5 eV,^23,24^ depending directly on the oxidization degree of the graphene and the species of the oxygen groups. However, solo GO exhibits weak photocatalytical activity. Combining with other semiconductors could improve their property and prepare excellent photocatalysts. The pioneering work of Zhang *et al.*^25^ on chemically bonded TiO_2_ (P25)–graphene composite photocatalyst was firstly reported. They discovered high photocatalytical performance of the nanocomposite containing GO and TiO_2_ (P25). Afterwards, there are many researches on the preparation of graphene–TiO_2_ composite^26–28^ and its wide application such as photocatalytic bactericide,^29^ hydrogen evolution,^30^ or dye-sensitized solar cell.^31^ Such kind of composites owns at least two advantages: (1) controllable reduction of the GO nanosheets incorporated in the composition by using UV irradiation and (2) enhanced photocatalytic activity of TiO_2_ thin films for higher efficiency of solar light irradiation. However, all the previous report about photocatalysts were usually powder and amorphous, which was difficult to handle and restricted its usage in practical applications.

In this work, we report a novel synthesis of TiO_2_ NRAs on Si, quartz and F-doped SnO_2_ (FTO) to form self-standing structures. The photocatalyst is much easier to recycle and extends its application. Besides, we tried to introduce Ag, Ag_2_S and GO together according to the effects described in above description and prepare a unique photocatalyst with high activity. Based on our recent work on GO synthesis,^32^ a rational design of GO/Ag/Ag_2_S–TiO_2_ nanocomposite is proposed for highly efficient photocatalysis under visible light irradiation. In this regard, the effect of different combination among Ag NPs, Ag_2_S NPs, GO and TiO_2_ NRAs on degradation efficiency and photoelectrocatalytic property are discussed briefly.

## Experimental methods

### Synthesis of TiO_2_ NRAs

The inclined TiO_2_ NRAs were deposited by glancing angle deposition technique (GLAD) technique on different substrates and annealed as previously reported.^33^ Before the deposition, the chamber was evacuated to a vacuum level above 1 × 10^−8^ Torr. During deposition, the vapor flux incident angle was set to ∼86° off the surface normal to the substrates. And the deposition rate (∼0.75 nm s^−1^) and height of the NRs were monitored by a quartz crystal microbalance. Ti films were oxidized in a tube furnace in order to obtain TiO_2_ NRAs. The Ti films were heated up to 400 °C for 2 h at a ramp of 5 °C min^−1^ under oxygen environment to obtain crystalline nanostructures for high photocatalytic activity.

### Ag_2_S NPs deposition on TiO_2_ NRAs

Ag_2_S NPs were deposited on TiO_2_ NRs through successive ion layer adsorption and reaction (SILAR) method with slight modification as described in our previous work.^15^ Firstly, TiO_2_ NRAs were immersed in 0.1 mg mL^−1^ AgNO_3_ solution for 30 s, followed by rinsing with DI water and then immersed in Na_2_S solution (12 mg mL^−1^) for 30 s. After this, they were rinsed in DI water for several times. This SILAR process was repeated for several cycles until the desired quantity of decorated nanocrystallites was achieved.

### Ag nanoparticles deposition on TiO_2_ NRAs

Ag NPs were deposited using the same method. The NRs were immersed in 0.1 mg mL^−1^ AgNO_3_ solution for 60 s, followed by rinsing with DI water and then immersed in NaBH_4_ solution (1.0 mg mL^−1^) for another 60 s, after which the resultant was rinsed with DI water for several times. This SILAR process was repeated for several cycles until the desired quantity of metallic nanocrystallites was achieved.

### Ag/Ag_2_S NPs deposition on TiO_2_ NRAs

The TiO_2_ NRAs were alternately coated with Ag and Ag_2_S NPs respectively for specific times. To determine the optimal loading amounts of Ag and Ag_2_S NPs decoration, the orthogonal experimental design was used, and results were shown in Table S1† (5 μM MO under visible light).

### Combination with GO

**Fig. 1:**
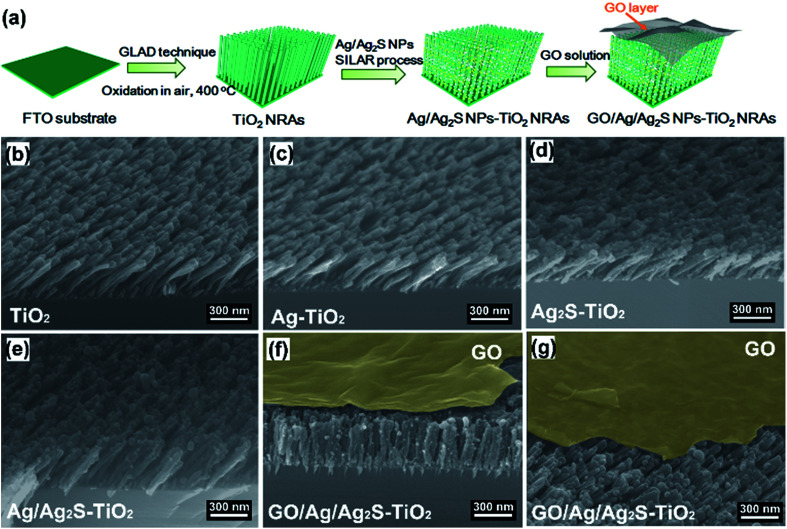
(a) Schematic illustration of the preparation of GO/Ag/Ag_2_S–TiO_2_ nanorod arrays (NRAs); scanning electron microscope (SEM) images of the different samples: (b) TiO_2_ NRAs; (c) Ag–TiO_2_ NRAs; (d) Ag_2_S–TiO_2_ NRAs; (e) Ag/Ag_2_S–TiO_2_ NRAs; (f) GO/Ag/Ag_2_S–TiO_2_ NRAs; (g) cross-section of sample GO/Ag/Ag_2_S–TiO_2_ NRAs.

GO was prepared *via* modified Hummers method. Typically, 5 g natural graphite flakes (Sigma Aldrich) and 3.0 g NaNO_3_ was added to a 1000 mL beaker containing 150 mL concentrated sulphuric acid in an ice-bath and stirred for 1 h. Then 20 g KMnO_4_ was gradually added while stirring, keeping the temperature below 30 °C. The mixture was stirred at room temperature for 24 h. Next, the beaker was placed in an ice bath and 300 mL DI water slowly added to the beaker, keeping the reaction temperature below 98 °C. After that, 30 mL H_2_O_2_ (30%) was added to the end reaction. Once H_2_O_2_ was added, the colour of the suspension turned bright yellow. The suspension was stirred for 30 min and then centrifuged at 8000 rpm to remove large flakes, then washed with 10% HCl solution, followed by washing with DI water and a three-week dialysis. Then Ag/Ag_2_S–TiO_2_ NRs with substrates were immersed in this solution for few seconds. The samples were dried in a muffle furnace at 50 °C for 2 hours to obtain the GO/Ag/Ag_2_S–TiO_2_ NRAs photocatalyst. GO–TiO_2_ NRAs, GO/Ag–TiO_2_ NRAs, GO/Ag_2_S–TiO_2_ NRAs were prepared in the similar way. The typical procedure were illustrated in Fig. 1(a).

### Materials characterization

The morphology and nanostructure of the fabricated samples were examined by field-emission scanning electron microscope (FE-SEM, JEOL-7001F), high-resolution transmission electron microscope (HRTEM, JEOL-2011) and Raman spectroscopy (LABRAM HR800, excitation wavelength of 633 nm), respectively. A Rigaku 2500 X-ray diffractometer was used to investigate the crystallographic characteristics of samples. The optical properties of samples were examined by a UV-vis spectrometer (Perkin Elmer Lambda 35) in a wavelength range from 400 nm to 900 nm at room temperature. The photoluminescence (PL) spectra were recorded by using Raman spectrometer (LabRAM ARMIS) with a 325 nm excitation. X-ray photoemission spectra were recorded by using ESCALAB 250Xi (Thermo Fisher). Monochromatized Al Kα was employed as anode. Survey spectra of all the samples were recorded from 0 eV to 1350 eV (binding energy) with each step at 1.0 eV, while the spectra for every specific element were collected with 0.05 eV/step. Mass Spectrometer (MS) analysis was performed with equipment of Thermo Scientific Q Exactive mass spectrometer.

### Photocatalytic degradation of crystal violet

The photocatalytic activity of different samples was evaluated by the photodegradation of crystal violet (CV) under visible light at ambient temperature. The sample on quartz substrate (15 mm × 15 mm) was placed in a quartz cell containing 5 mL of CV (5 μM) solution. Prior to light irradiation, the photocatalyst was immersed in a CV solution in dark for 30 min to reach an adsorption/desorption equilibrium, and Xe lamp with an ultraviolet filter was turned on for different time spans. After that, the concentration of CV was monitored using UV-vis spectroscopy at 584 nm. The degradation efficiency was calculated by (1 − *C*/*C*_o_) × 100%.

### Electrochemical measurements

The steady sate current density and electrochemical impedance spectroscopy (EIS) measurements were carried out in a three-electrode-cell controlled by an electrochemistry workstation (CHI 660D, Chenhua instrument). The nanostructured films were used as a working electrode with an exposed area of 1.5 cm^2^ in 0.1 M KOH solution (pH = 13.0). An Ag/AgCl electrode (saturated KCl) and Pt sheet were used as the reference and counter electrodes, respectively. In this paper, all the reported potentials refer to the Ag/AgCl electrode. Before the electrochemical measurements, cyclic voltammetry tests were scanned for several cycles to confirm the stability of the samples. EIS spectra were recorded from 0.1 Hz to 10^5^ Hz at open circle potentials for all the samples under visible light irradiation (>420 nm). Photocurrent densities with bias in the range of −0.8–0.0 V were measured with and without the visible light.

## Results and discussions

### Characterization of photocatalysts

Fig. 1(b–g) show the scanning electron microscope (SEM) images of all the prepared samples. It can be seen that these TiO_2_ NRAs regularly aligned on Si substrate having diameters of ∼50 nm and lengths of ∼200 nm (Fig. 1(b)). Ag and Ag_2_S NPs distributes randomly on TiO_2_ NRAs surface (Fig. 1(c–e)). GO of the sample GO/Ag/Ag_2_S–TiO_2_ NRAs, spreads smoothly like a blanket and covers Ag/Ag_2_S–TiO_2_ NRAs, which shows the connection of every single NRs to each other and with also some Ag and Ag_2_S NPs (Fig. 1(f), (g)).

**Fig. 2:**
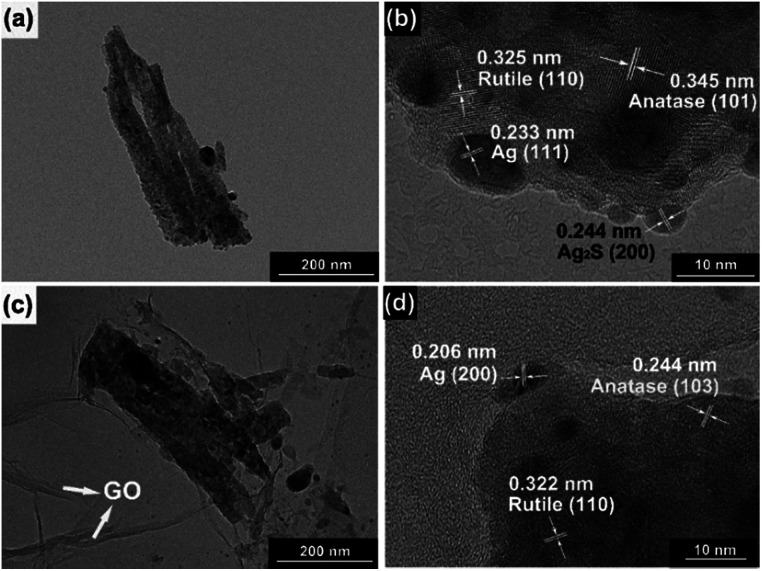
Transmission electron microscopy (TEM) images and high-resolution TEM (HRTEM) images of (a, b) Ag/Ag_2_S–TiO_2_ NRAs; (c, d) GO/Ag/Ag_2_S–TiO_2_ NRAs.

Moreover, transmission electron microscopy (TEM) images presented in Fig. 2 indicate Ag and Ag_2_S NPs were separately dispersed on the surface of TiO_2_. Their average sizes were about ∼4 nm, having a regular elliptical shape. According to the measurement of lattice fringes, *d* = 0.233 nm, 0.244 nm, 0.345 nm and 0.325 nm match well with the crystallographic planes of Ag (111), Ag_2_S (200), anatase (101) and rutile (110), respectively. The formation of metal–semiconductor nanojunctions, including Ag–TiO_2_ NRAs, could be favorable for interfacial charge transfer among the three components, enhancing photocatalytic activities of the composites. In addition, the existence of anatase–rutile heterojunction in the NRAs could help the rutile particles to efficiently collect photon-induced electrons from the anatase particles to reduce the carrier recombination.^34^

**Fig. 3:**
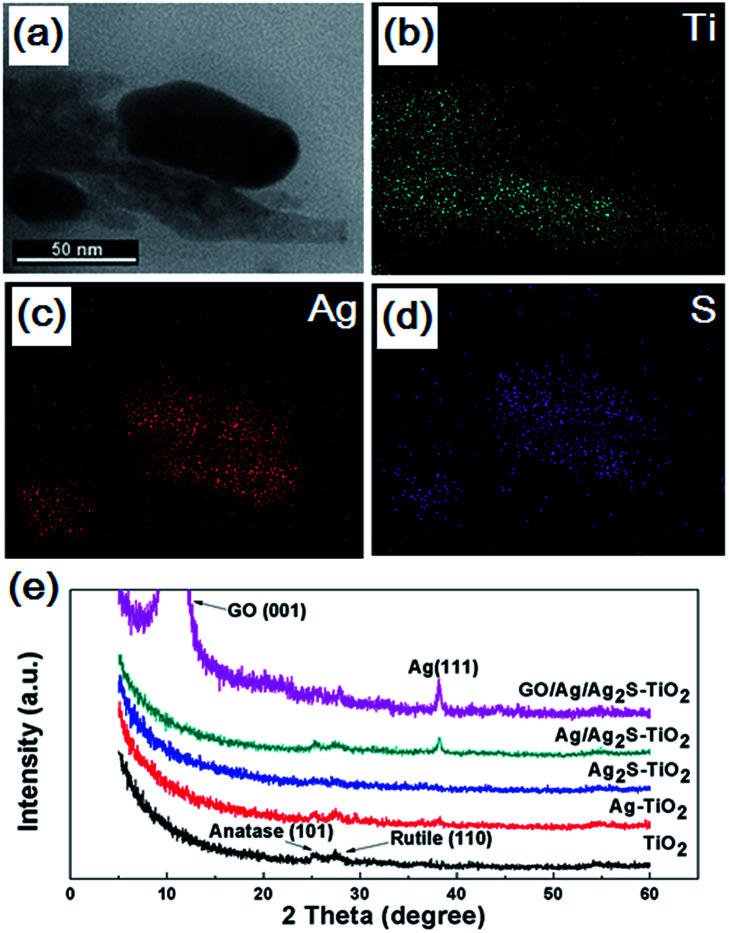
Morphology (a) and energy dispersive X-ray spectroscopy (EDX) elemental mapping of Ag/Ag_2_S–TiO_2_ NRAs sample: (b) Ti; (c) Ag; (d) S; (e) X-ray diffraction (XRD) patterns of the TiO_2_ NRAs, Ag–TiO_2_ NRAs, Ag_2_S–TiO_2_ NRAs, Ag/Ag_2_S–TiO_2_ NRAs and GO/Ag/Ag_2_S–TiO_2_ NRAs.

The Ti, Ag and S energy dispersive X-ray spectroscopy (EDX) element mapping of GO/Ag/Ag_2_S–TiO_2_ NRAs are shown in Fig. 3(a–d). The existence of Ag, S, Ti elements can be confirmed. Moreover, it can be concluded that Ag_2_S NPs adhere on the surface of TiO_2_ nanorods. X-ray diffraction (XRD) patterns of different samples are shown in Fig. 3(e). The spectra exhibited diffraction peaks at 25.5° and 27.6° corresponding to the (101) crystal planes of the anatase phase TiO_2_ (JCPDS no. 21-1272) and (110) crystal planes of the rutile phase TiO_2_ (JPCDS no. 21-1276). Beside this, the diffraction peak at 38.2° of Ag (111) (JCPDS no. 04-0783) can be found in the XRD patterns of Ag–TiO_2_ NRAs, Ag/Ag_2_S–TiO_2_ NRAs, and GO/Ag/Ag_2_S–TiO_2_ NRAs. However, characteristic peaks of Ag_2_S are very weak in all the samples. The intensive peak at 10.3° can be assigned to the (001) diffraction peak of graphene oxide. The full XRD spectrum of GO/Ag/Ag_2_S–TiO_2_ NRAs is shown in Fig. S1 as ESI.†

**Fig. 4:**
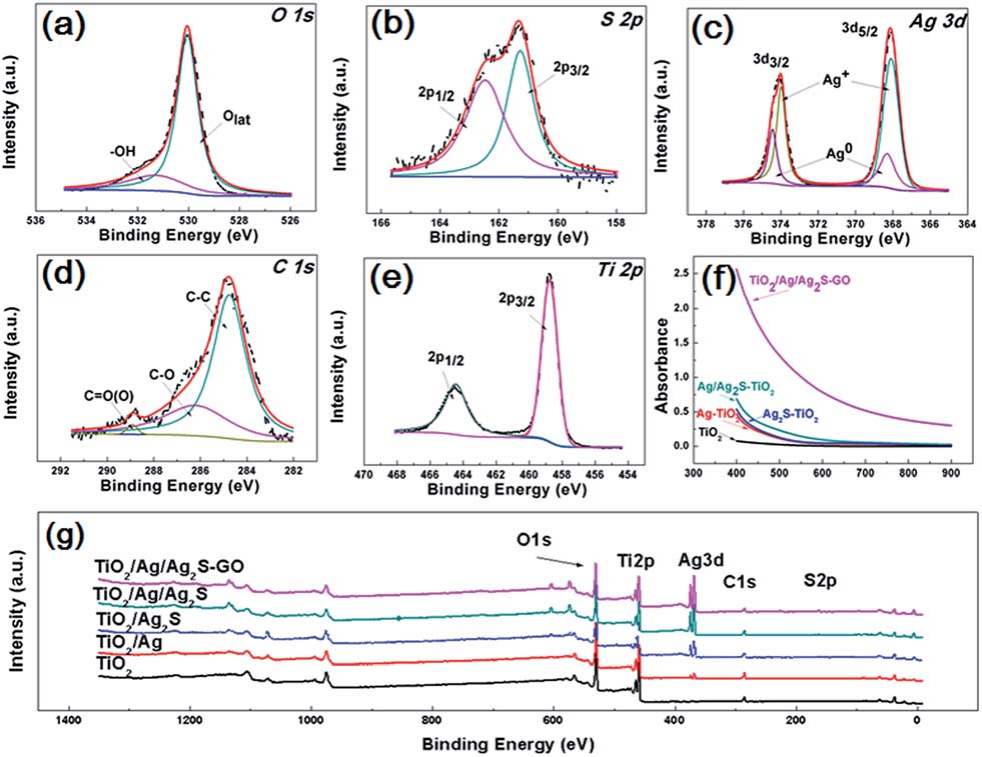
X-ray photoelectron spectroscopy (XPS) spectra of all samples: (a) O 1s; (b) S 2p; (c) Ag 3d; (d) C 1s; (e) Ti 2p; (g) survey of GO/Ag/Ag_2_S–TiO_2_ NRAs; (f) diffusion reflectance UV-vis spectra of TiO_2_ NRAs, Ag–TiO_2_ NRAs, Ag_2_S–TiO_2_ NRAs, Ag/Ag_2_S–TiO_2_ NRAs and GO/Ag/Ag_2_S–TiO_2_ NRAs.

Fig. 4(a–e) are the O 1s, S 2p, Ag 3d, C 1s, Ti 2p X-ray photoelectron spectroscopy (XPS) fine scan spectra of GO/Ag/Ag_2_S–TiO_2_ NRAs. XPS surveys of all samples are shown in Fig. 4(g). The O 1s spectrum can be fitted by two peaks: main peak located at the binding energy 530.0 eV which can be attributed to the lattice ‘O’; another one located at 531.3 eV referring to the hydroxyls or water adsorbed on the surface of the nanostructure. S 2p spectrum includes doublets containing S 2p_3/2_ and S 2p_1/2_ locating at 161.2 and 162.5 eV respectively, which can be assigned to S^2−^.^35^ Ag 3d spectra can be deconvoluted into two sets of doublet (Ag 3d_5/2_ and Ag 3d_3/2_ with spin orbit splitting of 6.1 eV (ref. 36)): Ag 3d_5/2_ peaks at 368.3 eV for metallic Ag and 368.1 eV for Ag^+^.^37^ C 1s spectra consist of the peaks at 284.9 eV, 286.3 eV and 288.9 eV, which are contributed by C–C; C–O and C=O bonds from deposited GO.^38^ Ti 2p spectrum including doublets of Ti 2p_3/2_ and Ti 2p_1/2_ at binding energies 458.8 and 464.4 eV respectively confirm the presence of Ti^4+^ cations in TiO_2_.

Diffusion reflectance UV-vis spectra of all typical resultants are shown in Fig. 4(f), which are fitted with Kubelka–Munk function. All the samples exhibit the absorption band around 400–900 nm. The spectra of Ag–TiO_2_ NRAs, Ag_2_S–TiO_2_ NRAs and Ag/Ag_2_S–TiO_2_ NRAs demonstrate obvious enhancement on light absorption in the visible region compared with TiO_2_ NRAs, implying the addition of Ag and Ag_2_S can increase the adsorption efficiency for TiO_2_. However, the surface plasmon resonance (SPR) absorption band is hardly observed in the spectrum of Ag–TiO_2_ NRAs because of the low imaginary part of the dielectric function of Ag. It can be clearly seen that the absorbance of GO/Ag/Ag_2_S–TiO_2_ NRAs is remarkably enhanced, which further confirms the contribution of GO for visible light utilization.

### Photodegradation of CV

To evaluate the effect of composite decoration on the photocatalytic activity of TiO_2_, the photodegradation of CV was carried out under visible light irradiation. For comparison, we also tested Ag/Ag_2_S–TiO_2_ NRAs, Ag–TiO_2_ NRAs, Ag_2_S–TiO_2_ NRAs and TiO_2_ NRAs. All these samples combined with GO were also tested. As shown in Table 1, after 120 min illumination under visible lights, neither TiO_2_ NRAs nor GO-TiO_2_ NRAs exhibited any activity for the CV degradation due to nonabsorbance of TiO_2_ in visible light range, while 16.32%, 13.51% and 28.92% of CV were degraded by Ag–TiO_2_ NRAs, Ag_2_S–TiO_2_ and Ag/Ag_2_S–TiO_2_ NRAs. These results indicated that both Ag and Ag_2_S NPs could be excited by visible light. Ag NPs transform the irradiation photons energy into localized SPR oscillations and hot electrons move quickly to other part of nanostructured electrode.^39–42^ Higher activity of Ag/Ag_2_S–TiO_2_ NRAs could be attributed to the photoexcited Ag_2_S semiconductor and plasmon-induced Ag NPs. Moreover, further enhancement of photocatalytic activities was observed after the introduction of GO. No matter what kind of combinations, after covering with GO, the photocatalytic degradation efficiencies were improved by 200–300%. With the combination of GO/Ag/Ag_2_S–TiO_2_ NRAs, the degradation efficiency of CV reached maximum to 80.01%. Therefore, GO played an important role in the enhanced activity of Ag/Ag_2_S–TiO_2_ NRAs.

**Table 1:** Degradation efficiency of CV under 120 min-visible light irradiation

Sample	TiO_2_	GO–TiO_2_	Ag–TiO_2_	GO/Ag–TiO_2_
Degradation efficiency (%)	0	0	16.32	47.00
Sample	Ag_2_S–TiO_2_	GO/Ag_2_S–TiO_2_	Ag/Ag_2_S–TiO_2_	GO/Ag/Ag_2_S–TiO_2_
Degradation efficiency (%)	13.51	31.77	28.92	80.01

**Fig. 5:**
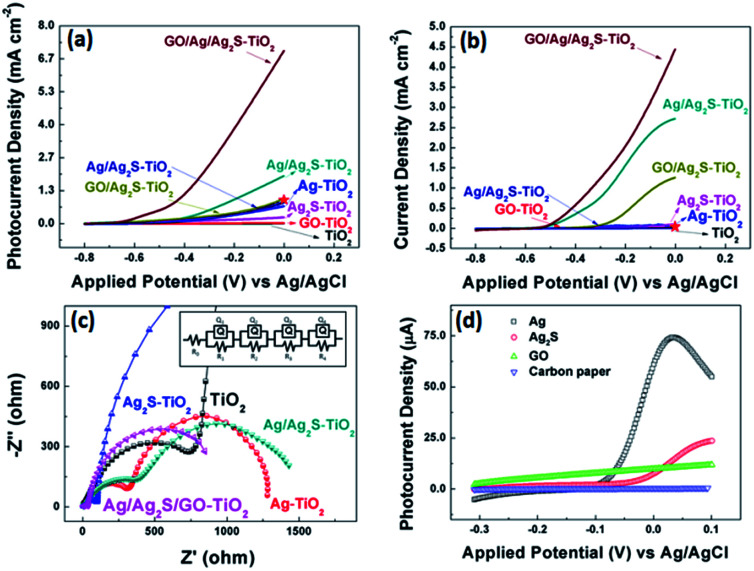
(a) *j*–*V* characteristics of all samples under visible light illuminations; (b) *j*–*V* characteristics of all samples without light illuminations. The red stars in (a, b) indicate the current density of TiO_2_/rGO/NiFe-LDH NRAs reported by Ning *et al.* under same voltage condition;^36^ (c) partial enlarged detail Nyquist plots under visible light at open circuit voltage of all decorated sample and the equivalent circuit for samples; (d) *j*–*V* characteristics for various combination of Ag, Ag_2_S and GO decoration on carbon paper under visible light illuminations.

Fig. 5 (a and b) show the comparison of the photocurrent density of different samples with light on/off under simulated solar irradiation. The photocurrent of pure TiO_2_ was zero, while the photocurrent density of Ag–TiO_2_ NRAs and Ag_2_S–TiO_2_ NRAs increased. And Ag/Ag_2_S–TiO_2_ NRAs raised to about 1.92 mA cm^−2^ at 0 V. After composited with GO, the photocurrent of GO/Ag/Ag_2_S–TiO_2_ NRAs was remarkably improved 10 times to 6.77 mA cm^−2^ at 0 V, which indicated that higher efficient separation of photogenerated carriers occurred. At the same condition, dark current density of GO/Ag/Ag_2_S–TiO_2_ NRAs (4.64 mA cm^−2^ at 0 V) was only 68% of that under visible light. Thus reasonable amount of GO in nanostructure could act as a sinker for photoinduced charge carriers, promoting charge separation to enhance the overall photocatalytic efficiency in contact with TiO_2_. The promotion could be contributed to the addition of p-type semiconductor of GO which has hollow sites.^14^ In the GO/Ag/Ag_2_S–TiO_2_ NRAs sample, Ag_2_S and TiO_2_ are both n-type,^43^ the introduction of GO will compose p–n junction in the sample, then the junction could enhance the current transferring through the electron and hole sites among the composites of the sample. Ning *et al.* have reported a core–shell heterostructure TiO_2_/rGO/NiFe-LDH NRAs which own an enhanced photoconversion efficiency (0.58% at 0.13 V *vs.* SCE) and photocurrent density (1.74 mA cm^−2^ at 0.6 V *vs.* SCE).^44^ For comparison, we plot current value at same voltage as red star on our PEC figures. Moreover, in site doped Au/TiO_2_ nanotube photoanode made by Wu.^45^ and GO-coated TiO_2_ nanoparticles fabricated by Kim *et al.*^46^ were all tested by photocurrent. The relative datas were organized in Table 2. Compared with recent results, our material's performance on separation of electrons and holes make a quite big progress.

**Table 2:** Comparison of recent work on photocurrent and relative excitement condition

Group/system	Photocurrent (mA cm^−2^)	Voltage (V)	Light resource	Electrolyte
Ning *et al.* TiO_2_/rGO/NiFe-LDH nanorods	1.74	0.6	150 W Xe lamp	0.5 M Na_2_SO_4_ solution
Wu *et al.* Au/TiO_2_ nanotubes	0.50	0	300 W Xe lamp	0.1 M Na_2_SO_4_ solution
Kim *et al.* GO-coated TiO_2_ nanoparticles	1.25	0	300 W Xe arc lamp	0.1 M HClO_4_ solution
This work	6.98	0	300 W Xe lamp	0.1 M KOH solution

EIS measurements for all samples were also conducted under visible light irradiation, as shown in Fig. 5(c) as the enlarged image of full spectra (see Fig. S2†). This method can characterize the charge-carrier migration. An equivalent circuit was used to fit the Nyquist plots as shown in the inset of Fig. 5(c) and simulated data were shown in Table S2.† All the hybrid nanostructured samples showed depressed semicircles at high frequencies compared with pure TiO_2_ counterparts. The reduced semicircles indicate diminished resistance of working electrodes, suggesting a decrease in the solid state interface layer resistance and the charge transfer resistance across the solid–liquid junction on the surface by forming hybrid structures of decorated TiO_2_ NRAs with GO.^47^ The *R*_0_ are the resistance of FTO substrate. *Q*_1_ and *R*_1_ represent the double layer on the reference electrode. The obtained Nyquist spectra show two arcs, the first arc (*R*_2_), at higher frequencies, is related to the interfacial resistance between TiO_2_ nanorod array and electrolyte. And the arcs at the intermediate frequencies represent the resistance (*R*_3_) of holes transferred to the electrolyte through surface states (such as Ag or Ag_2_S NPs) and the capacitance of surface states. For Ag/Ag_2_S–TiO_2_ NRAs, Ag NPs was deposited after Ag_2_S decoration.

However, based on HRTEM analysis, the Ag and Ag_2_S are separately attached on the TiO_2_ arrays surface. So, an extra resistance (*R*_4_) was added and can be described as electron or hole transfer between NPs of Ag and Ag_2_S. When layer of GO was coated, the *R*_4_ can also represents the transfer between the decorated TiO_2_ and GO. All simulated data are listed in Table S2.†^48^ Based on the results, it can be seen that the additions of Ag or/and Ag_2_S can decrease the *R*_2_ and *R*_3_, implying the reduced transferring resistance between the deposition substrate and TiO_2_. The extra introduction of GO can decrease the hole transfer resistance (*R*_3_) dramatically. By comparing the EIS data, GO/Ag/Ag_2_S–TiO_2_ NRAs are superior to other samples with smaller semicircles, suggesting a rapid transport of charge carriers and an effective charge separation, which is in agreement with the PEC analysis.

In the photocurrent measurements, the obtained currents tested under visible light include photocurrent for oxygen evolution reaction (OER), and electrochemical current caused by applied potential in OER process. So, we use current with light minus the one without light in order to investigate more specific on photo-catalysis progress. Then, the largest current value obtained on GO/Ag/Ag_2_S–TiO_2_ NRAs was used as reference. The currents of all the other samples were divided by the reference to calculate the relative values, which are listed in Table 3. According to data, it can be easily obtained that simply composing between Ag and Ag_2_S particles doesn't improve the photocatalytic performance so much. Only after the addition of GO layer, the activities of all system enhance remarkably, while the GO/Ag/Ag_2_S–TiO_2_ NRAs has maximum increment up to 7.7 times. GO layer could both enhanced transmission of charges and protect NPs from corrosion. What's more, to confirm the reaction mechanism and active reaction sites among all the possible composites, we deposited the single composite on carbon paper rather than TiO_2_ NRAs. Then the photocurrents of the prepared samples were recorded under the same test condition which are shown in Fig. 5(d). Basing on the tests, the onset potentials of Ag is most negative among all the prepared samples, implying that Ag has lowest OER overpotential among the four species.

**Table 3:** Current density percentage (excluded OER) of all samples at −0.3 V *vs.* Ag/AgCl

Sample	TiO_2_	GO-TiO_2_	Ag–TiO_2_	GO/Ag–TiO_2_
Percentage (%)	0	0.22	12.89	2.11
Sample	Ag_2_S–TiO_2_	GO/Ag_2_S–TiO_2_	Ag/Ag_2_S–TiO_2_	GO/Ag/Ag_2_S–TiO_2_
Percentage (%)	7.59	16.11	12.97	100.00

**Fig. 6:**
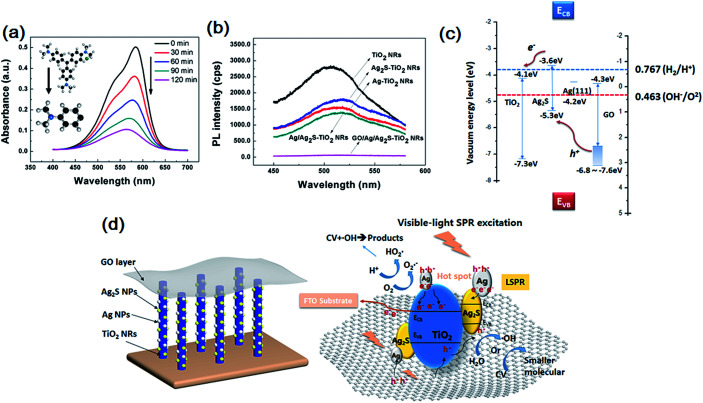
(a) UV-visible absorption spectra recorded during the catalytic degradation of CV over GO/Ag/Ag_2_S–TiO_2_ NRAs; (b) photoluminescence (PL) spectrum of single TiO_2_ NRAs, Ag–TiO_2_ NRAs, Ag_2_S–TiO_2_ NRAs, Ag/Ag_2_S–TiO_2_ NRAs and GO/Ag/Ag_2_S–TiO_2_ NRAs; (c) band edge positions of TiO_2_, Ag_2_S, Ag and GO; (d) photocatalytic process for GO/Ag/Ag_2_S–TiO_2_ NRAs under visible light.

The evolution of the CV solution's absorption spectra for GO/Ag/Ag_2_S–TiO_2_ NRAs are recorded during the whole reaction process every 30 min time point. From Fig. 6(a), the intensity of peak gradually declines with longer irradiation time which indicates reduced concentrate of dye concentrate. To analyse the products after reaction, mass spectrometer (MS) for CV in aqueous solution before and after prolonged irradiation (120 min) are also studied, shown in Fig. S3.† As standard CV molecular (*M* = 407.99) concerned, peak at *m*/*z* = 372.23 indicate CV molecular which equals to formula weight minus chloridion. When we infer product after reaction from *m*/*z* = 152.12, it is exactly coincident to 1/3 part of whole molecular that three C–C bonds of C atom in central broken up. The result means there is a single product after degradation reaction.

Photoluminescence (PL) technique is an effective way to study the efficiency of the charge carrier trapping, migration and transfer, as PL signals result from the recombination of photo-induced carriers. Fig. 6(b) presents the PL spectra of all test samples. The peaks at ∼515 nm can be attributed to the self-trapped excitations and the oxygen vacancies (*V*_o_) in TiO_2_.^49,50^ Due to the metallic particle decoration on TiO_2_, the PL peaks of TiO_2_ become weaker. The PL intensity of GO/Ag/Ag_2_S–TiO_2_ NRAs is much lower than that of other nanostructures, implying a lower recombination rate of photo-induced electron–hole pairs, and thus a better photocatalytic performance. While PL intensity of Ag–TiO_2_ NRAs, Ag_2_S–TiO_2_ NRAs and Ag/Ag_2_S–TiO_2_ NRAs are between TiO_2_ NRAs and GO/Ag/Ag_2_S–TiO_2_ NRAs which is corresponding with dye degradation results.

### Photocatalytic mechanism

Basing on above results, the photocatalytic mechanism of GO/Ag/Ag_2_S–TiO_2_ NRAs can be proposed as below. The valence band-edges of materials are shown in Fig. 6(c). In the composite, Ag NPs, Ag_2_S NPs (always n-type) and GO sheets (typical p-type) are active to visible light. The higher activity of GO/Ag/Ag_2_S–TiO_2_ NRs could be contributed to the photoexcited.

Ag_2_S semiconductor, plasmon-induced Ag NPs, effective electron–hole separation of GO and clever combination of all the composites. Under visible light irradiation, Ag nanoparticles are photoexcited owing to their SPR effect, and then charge separation is accomplished, where the electrons transfer from Ag nanoparticles to the TiO_2_ CB, leading to the generation of holes in Ag nanoparticles for oxidation of OH^−^. Meanwhile, Ag_2_S NPs and GO are also excited to form electrons and holes.^51^ Considering that the CB of Ag_2_S is more positive than that of TiO_2_,^52,53^ electrons generated from Ag_2_S also tend to transfer into the CB of TiO_2_. This facilitates the separation of electrons and holes. Besides, covering of GO layer remarkably enhances full-wavelength absorption from UV-vis spectra reported in Fig. 4(f), which accomplishes high-usage of visible light. Since work electrode surface is positively charged, excited electrons from GO are easier to move into TiO_2_. Then, vast holes tend to move directly into Ag NPs which consume holes quickly because of its lowest OER overpotential among the four species in the composite, resulting acceleration of the whole reaction. Thus Ag NPs is probably active OER reaction sites for the whole system. Ag nanoparticles also act as the charge transmission bridges, which can efficiently promote the separation of electron–hole pairs. Also, both Ag and Ag_2_S combination with TiO_2_ could narrow down its original band gap, it may enhance the adsorption efficiency of the composite.

Then electrons can either reduce the dye or can react with electron acceptors (O^2^ absorbed on the surface of Ti^3+^ or dissolved in water) to create superoxide radicals (O^2^˙^−^). Meanwhile, the resultant electron-deficient Ag particles can oxidize the organic molecule or react with OH^−^ to form hydroxyl radicals, OH˙. The process is demonstrated in Fig. 6(d). The co-decoration of Ag/Ag_2_S NPs and GO not only extends TiO_2_ to visible light region, but also increases the efficiency of charge separation and improves its photocatalytic efficiency.

## Conclusions

In summary, we obtained the procedure for preparation of the TiO_2_ NRAs on various substrates including FTO, Si and *et al.*, and fabricated successfully GO/Ag/Ag_2_S–TiO_2_ NRAs by using SILAR technique and impregnation method. By comparing with TiO_2_ NRAs, it exhibits remarkable improvement of photocatalytic activity for dye degradation and PEC performance. The enhanced photocatalytic activities through decoration of the NRAs are attributed to the synergy of matched band of Ag_2_S NPs, SPR effect of Ag NPs and rapid transport of charge carriers that improves charge separation of system. Our studies demonstrate that we can utilize photon in a wider range of solar spectrum to generate charge carriers for photocatalytic reactions through rational design of composite nanostructures. The combination of GO with traditional photocatalyst also provides a more effective way to harvest solar energy for decomposition reaction.

## Conflicts of interest

There are no conflicts to declare.

## Supplementary Material

RA-008-C7RA13501G-s001
