# Supplementary material for: Efficient photocatalysis with graphene oxide/Ag/Ag2S–TiO2 nanocomposites under visible light irradiation
Source: RSC Adv. 2018 Feb 5;8(11):5784–91. doi: 10.1039/c7ra13501g (PMC9078160; doi:10.1039/c7ra13501g)
Supplement: RA-008-C7RA13501G-s001 [file RA-008-C7RA13501G-s001.pdf]

Electronic Supplementary Material (ESI) for RSC Advances.
This journal is © The Royal Society of Chemistry 2018

Supporting Information for

Efficient photocatalysis with graphene oxide/Ag/Ag_2S-TiO_2 nanocomposites under visible light irradiation†

*Shuang Shuang,[a] Ruitao Lv,[b] Xiaoyang Cui,[a] Zheng Xie,[a, c] Jian Zheng [d] and Zhengjun Zhang [b]**

a. State Key Laboratory of New Ceramics and Fine Processing, School of Materials Science and Engineering, Tsinghua University, Beijing 100084, China

b. Key Laboratory of Advanced Materials (MOE), School of Materials Science and Engineering, Tsinghua University, Beijing 100084, China

c. High-Tech Institute of Xi'an, Xi'an 710025, China

d. Department of Chemistry, University of Oslo, Sem Sælands vei 26, 0371 Oslo, Norway

* Addresses correspondence to zjzhang@tsinghua.edu.cn (Z.J.Z.)

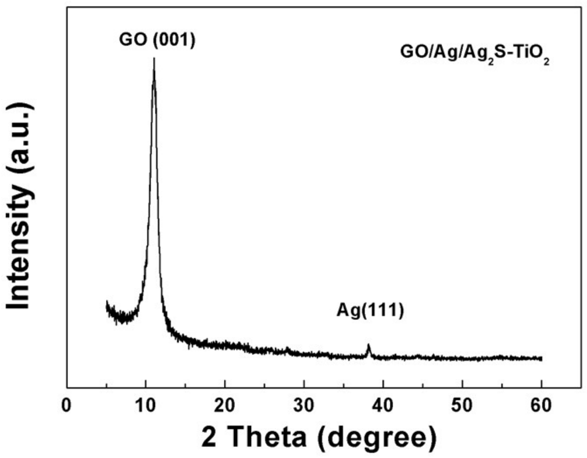

Figure S1. XRD spectrum of $GO/Ag/Ag_2S$-TiO_2 sample.

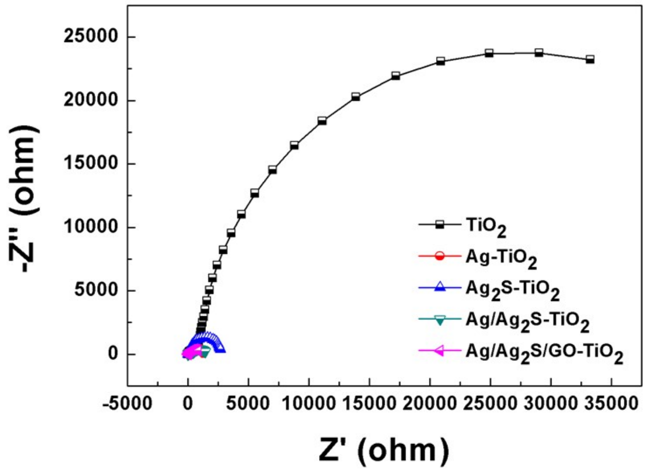

Figure S2. Full Nyquist plots under visible light at open circuit voltage of all decorated sample.

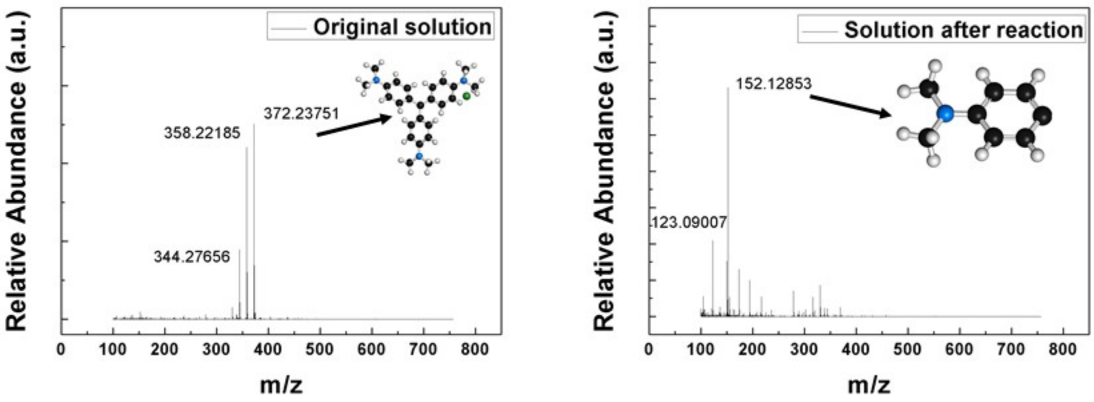

Figure S3. MS spectra of CV(aq), before and after prolonged irradiation for GO/Ag/Ag_2S-TiO_2 NRAs and related molecular structures.

Table S1 Degradation efficiency of different Ag and Ag_2S decorated cycle numbers combination (5μM MO under visible light)

**Ag_2S\Ag（cycles）**	**5**	**10**	**15**	**20**
**10**	0.228	0.239	0.201	0.192
**20**	0.161	0.259	0.202	0.206
**30**	0.149	0.214	0.190	0.170
**40**	0.143	0.176	0.162	0.140

Table S2 The calculated values of all elements through fitting of the experimental impedance spectra based upon the proposed equivalent circuit in Figure 5(d)

**Sample**	**R_0**	**Q_1**		**R_1**	**Q_2**		**R_2**	**Q_3** **CPE2-P**		**R_3**	**Q_4**		**R_4**
		Y_1	n_1		Y_2	n_2		Y_3	n_3		Y_4	n_4	
**TiO_2**	2.55	3.19×10^{-7}	0.917	15.5	2.40×10^{-6}	0.773	809	2.14×10^{-5}	0.928	5390	--	--	--
**Ag- TiO_2**	2.91	4.43×10^{-7}	0.853	10.8	1.18×10^{-5}	0.727	337	1.24×10^{-4}	0.948	958	--	--	--
**Ag_2S-TiO_2**	2.95	6.90×10^{-7}	0.825	11.8	8.54×10^{-6}	0.790	89.3	9.37×10^{-5}	0.976	2685			
**Ag/Ag_2S-TiO_2**	3.11	2.18×10^{-7}	0.927	9.41	2.86×10^{-5}	0.613	454.9	1.51×10^{-4}	0.919	747	2.79×10^{-3}	0.680	696
**GO/Ag/Ag_2S-TiO_2**	3.05	3.92×10^{-7}	0.871	21.2	1.34×10^{-4}	0.928	674	3.87×10^{-4}	0.570	18.6	1.60×10^{-3}	0.760	1011
